# Changes in the Linear Relationship between Muscle Contraction Intensity and Muscle Hardness after Rectus Femoris Muscle Strain

**DOI:** 10.1155/2019/7813217

**Published:** 2019-11-25

**Authors:** Takayuki Inami, Takuya Shimizu, Tomoaki Osuga, Takaya Narita, Norikazu Hirose, Mitsuyoshi Murayama

**Affiliations:** ^1^Institute of Physical Education, Keio University, 4-1-1, Hiyoshi, Kanagawa 223-8521, Japan; ^2^Graduate School of Health and Sport Sciences, Chukyo University, 101, Tokodate, Toyota City, Aichi 470-0348, Japan; ^3^Osuga Clinic Orthopaedics, 117, Takegaya, Tokai City, Aichi 477-0032, Japan; ^4^Department of Sports Technology, Toin University of Yokohama, 1614 Kurogane-cho, Aoba-ku, Yokohama, Kanagawa 225-8503, Japan; ^5^Faculty of Sport Sciences, Waseda University, 2-7-5, Higashi-Fushimi, Tokyo 202-0021, Japan

## Abstract

**Objective:**

Joint torque differences between healthy and rehabilitated legs are often measured as a clinical index of recovery from muscle strain injury. Unfortunately, it should be noted that this is a questionable evaluation measure of the muscle after injury because it is a composite value including related cooperating muscles. Meanwhile, the use of ultrasound elastography for the measurement of individual muscle mechanical properties (i.e., muscle hardness) has recently expanded. The purpose of this study was to examine, using ultrasound elastography, the differences in the linear relationship between muscle contraction intensity and muscle hardness during knee extension in athletes who had recovered from grade II rectus femoris muscle strain injury through comparison of the healthy and rehabilitated legs.

**Methods:**

Six athletes participated. Rectus femoris muscle hardness, determined during isometric contraction at 10%, 20%, 30%, and 40% of maximum voluntary contraction, was evaluated using ultrasound strain elastography.

**Results and Conclusion:**

The results indicated that for the healthy legs, the strain ratios, as indicated by muscle hardness, decreased linearly (became harder) with contraction intensity, but the strain ratios for the rehabilitated legs decreased nonlinearly. These results show the danger of judging the recovery period using only the difference between healthy and rehabilitated muscle strengths and the importance of evaluating individual muscles.

## 1. Introduction

Quadriceps muscle strain injuries frequently occur during sporting events [1, 2], and the rectus femoris is the most frequently injured quadriceps muscle [3]. A prospective study reported muscle strength as a risk factor, with differences in muscle strengths between the injured and healthy legs being indicators of recovery [4]. However, joint torque, as a measure of muscle strength, is a composite value derived from related cooperating muscles (i.e., the vastus lateralis, vastus intermedius, and vastus medialis muscles). Hence, it should be noted that joint torque measurement is questionable as an independent measure of recovery of individual muscles.

Recently, ultrasonic elastography has used for the measurement of individual muscle mechanical properties (e.g., muscle hardness) [5]. Muscle hardness measured using ultrasound strain elastography (USE) has been reported to be linearly related to muscle contraction intensity up to a moderate level of contraction, and USE is attracting attention as a method for estimating individual muscle strength (based on individual muscle hardness) [6]. This linear relationship is hypothesized to be applicable to both leg muscles if there is no difference in the joint torque of the legs even if the injured muscle has already recovered. However, this relationship has not been specifically investigated.

The present study is aimed at examining the differences in the above relationship by comparing changes in muscle hardness during knee extension between legs with rectus femoris strain injuries and healthy contralateral legs.

## 2. Case Presentation

The participants (three men and three women) were track and field athletes of 100 or 400 m hurdles who visited the same orthopedic clinic. This study had a retrospective study design, and participants were selected among the clinic patients. The participants have been diagnosed using MRI with grade II rectus femoris muscle strain injuries at the time of injury by an orthopedic surgeon in 50% of the line connecting the greater trochanter and the knee lateral joint space (i.e., muscle belly), based on a previous severity classification study [7]. The measurement later described was performed at the time when rehabilitation by an orthopedic surgeon and physiotherapist was completed. The participants' characteristics are shown in Table 1. This study was approved by the local institutional ethics committee and conducted in accordance with the Declaration of Helsinki (2011-025).

### 2.1. Isometric Contraction Strength

Similar to a previous study design [6], participants were seated on dedicated chair, with their hip and knee joints fixed at 90° and their ankles and trunks secured with nonelastic belts. Each participant performed a maximum voluntary contraction isometric knee extension against a load cell (LU-100KSB34D, Kyowa Electric Instruments, Japan), which was connected to a strain amplifier (F420, Unipulse, Tokyo, Japan) and an AD converter (LX-10, TEAC, Tokyo, Japan). The force was recorded using a data recorder (D252, Unipulse) connected to a personal computer with a 1000 Hz sampling frequency and a low-pass filter (cutoff frequency, 10 Hz). Based on the maximum voluntary contraction (at 10, 20, 30, or 40%), the target force was displayed on a computer screen, and each participant was instructed to maintain that force for 5 s with a 3 min rest between different contraction intensities.

### 2.2. Muscle Hardness Measurement Using USE

An ultrasound system with elastography function (Ascendus; Hitachi Medical, Tokyo, Japan) was used. A linear array transducer (EUP-L65, Hitachi, Japan) was placed perpendicularly on the rectus femoris muscle belly at the mid-thigh level (i.e., injured site), based on a line connecting the great trochanter and lateral femoral condyle. An acoustic coupler (EZU-TECPL1, Hitachi) was used as a reference that was separately attached to the transducer. Based on a previous study [6], the acoustic coupler is made of elastomer resin and its elastic modulus is a constant 22.6 kPa. The investigator manually and repeatedly pressed the transducer and acoustic coupler against the muscle, while monitoring the consistency of the pressing force strain on the ultrasonic apparatus. The ultrasound image was recorded for 5 s, with the first and last seconds excluded; up to 45 elastography images (3 s) were recorded. From these images, 10 were selected based on the clarity of the colors of the muscle tissue and acoustic coupler and the pressing force strain graph showing a -0.7 to 0.7 range during the rhythmical compression-relaxation cycles, according to a previous study [6]. If more than 10 images were usable, 10 were randomly selected.

Based on a previous study [6], one rectangular region of interest was set in the region showing the coupler (4 mm long × 30 mm wide). Other regions of interest were set along the rectus femoris muscle. After setting the two regions of interest, the strain within each was automatically calculated by a built-in software, and the strain ratio was calculated using the following formula [6]:

(1) $$\begin{array}{c} \frac{E\text{ muscle}}{E\text{ acoustic coupler}}=\frac{\sigma \text{ muscle}/\varepsilon \text{ muscle}}{\sigma \text{ acoustic coupler}/\varepsilon \text{ acoustic coupler}}, \end{array}$$

where *E* is Young's modulus, *σ* is the stress, *ε* is the strain, and *E* = *σ*/*ε* is Hooke's law.

When the strain ratio is 1, the muscle hardness is identical to the reference; as stated in previous studies [5, 6], smaller strain ratios indicate harder muscle. The average value for the 10 images was used as the representative strain ratio.

### 2.3. Statistical Analysis

A one-way repeated measure analysis of variance (ANOVA), with subsequent multiple comparisons performed using a Bonferroni post hoc test, was used to compare the strain ratios obtained for the four isometric contraction intensities. A two-way repeated measures ANOVA was used to compare the contraction intensity-strain ratio relationship between the healthy and rehabilitated legs. The results are shown as means ± standard deviation; *p* < 0.05 was statistically significant.

## 3. Results

The damaged leg of all participants was the leg involved in hurdle take off (i.e., not the lead leg). The mean joint torque of the healthy and rehabilitated legs was 183.6 ± 23.4 and 177.5 ± 23.1 Nm, respectively; the healthy and rehabilitated legs had no significant difference.

**Figure 1:**
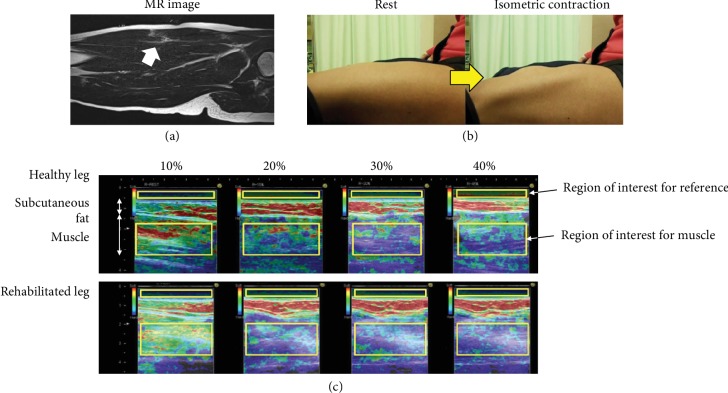
(a) An MR T1 image of the rectus femoris muscle. (b) The aspect of the rectus femoris muscle during rest and contraction. (c) Strain elastographic images in healthy and rehabilitated legs related to isometric contraction intensity (10%, 20%, 30%, and 40% of maximal voluntary isometric contraction) from one subject. The strain ratios are shown by two yellow rectangular boxes, with the upper box showing the reference and the lower box showing the muscle that is the region of interest. The strain ratio has been calculated using the built-in software. The color in the region of interest (square) changes from yellow/green to blue.

**Figure 2:**
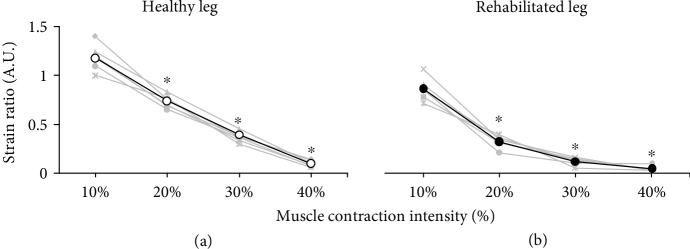
Changes in strain ratio relative to isometric contraction intensity (10%, 20%, 30%, and 40% of maximal voluntary isometric contraction) for the healthy (a) and rehabilitated (b) legs of six subjects. Each subject is indicated by a different symbol, but same symbols are used for the healthy and rehabilitated legs. For each graph, the average values of the six subjects are shown using open (healthy legs) and closed (rehabilitated legs) circles. ^∗^Significantly (*p* < 0.01) different from the 10% of maximal voluntary isometric contraction. A.U.: arbitrary units.

**Figure 3:**
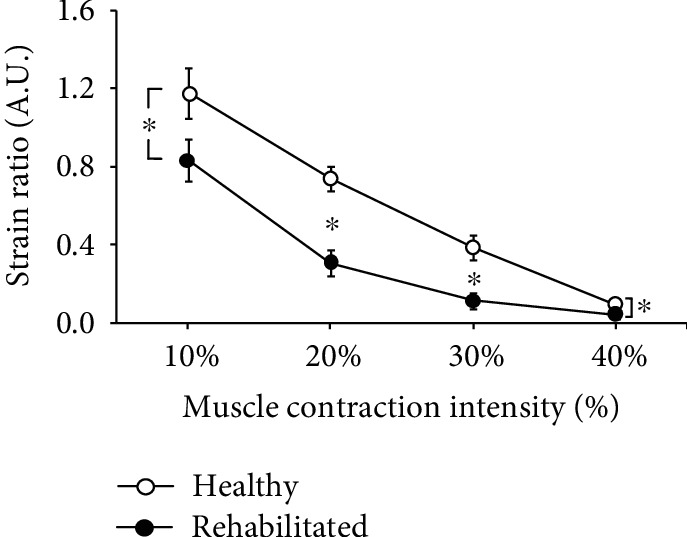
Comparison of the average strain ratios for healthy and rehabilitated legs. A significant interaction effect was found for the relationship between contraction intensity and strain ratio between legs. ^∗^Significant (*p* < 0.01) difference between legs. A.U.: arbitrary units.

Figure 1(a) and (b) show the MR T1 image and the aspect of the rectus femoris during contraction. In addition, Figure 1(c) shows an example of the relationship between contraction intensity and elastographic images. The average strain ratio for the healthy leg decreased linearly with increased contraction intensity (*p* < 0.05) (Figure 2). However, the average strain ratio for the rehabilitated legs decreased nonlinearly with increased contraction intensity. Significant interaction differences were found between the healthy and rehabilitated legs at all contraction intensities (*p* < 0.05) (Figure 3).

## 4. Discussion

The results of this study did not support the hypothesis that joint torque is the same between healthy and rehabilitated legs and that both demonstrate similar linear relationships.

The relationship between contraction intensity and muscle hardness of the healthy leg supported the results of a previous study [6]. Some reports have confirmed a linear increase in muscle contraction intensity-related muscle hardness in healthy legs [8, 9]. According to previous studies [6, 9], the increase in muscle hardness is mainly due to the active state of cross-bridges, with the number of cross-bridges determining muscle hardness during contractions involving up to moderate intensities [8].

The mean joint torque of the rehabilitated legs was the same as that of the healthy legs, but no linear relationship was observed between muscle hardness and contraction intensity. According to Cross et al. [3], more than half of the cases of rectus femoris muscle strains involve the parenchyma, as supported by the findings in the present study. Furthermore, the damaged tissue in the muscle parenchyma is highly likely to be fibrosis [10]. The MR T1 image and elastography images (Figure 1) in the present study also confirmed that the muscle tissue was depressed and that there was fibrosis in all participants, suggesting the possible presence of abnormal cross-bridges in the rehabilitated legs. In particular, regarding the presence of fibrosis, as the regions with fibrosis were included in the ROI of the elastography analysis, it may have affected the increasing muscle hardness. On the other hand, regional differences in muscle activities have been reported for rehabilitated legs, assessed using surface electromyography [10], during knee extensions in old rectus femoris muscle strain cases; thus, muscle activity in the injury region may be different compared with that of the healthy leg. Conversely, the advantage of USE is that it can secure a relatively large ROI, and it has been reported that the strain ratio is not influenced by depth [11]. Thus, the findings of this study provide information regarding changes in the mechanical properties including the deep layer that cannot be revealed by surface electromyography.

Interestingly, the damaged leg of all participants in this study was, coincidentally, the leg involved in hurdle take off. We speculate that when a hurdler runs over a hurdle, the hip joint extension on the taking-off side leg is of a greater magnitude than during other phases, so that the stride becomes larger, and consequently, the rectus femoris muscle is stretched more. We tracked longitudinally the performance before and after injury in one participant as an example and found that the season best of 100 m hurdle was 13.2 seconds before the injury but significantly increased to 14.9 seconds immediately after the injury and returned to 13.8 seconds 3 years later. Although the strain ratio is only a predictor of muscle strength, these results could well be an important finding that may have implications for muscle function and reinjury susceptibility.

The present study shows the danger of judging the recovery period based only on differences between healthy and injured joint torque and the importance of evaluating individual muscles.

## Figures and Tables

**Table 1:** Characteristics of the subject.

	Men (*n* = 3)	Women (*n* = 3)
Age (years)	25.6 ± 0.9	28.8 ± 3.7
Height (cm)	172.6 ± 3.5	159.6 ± 3.2
Body mass (kg)	66.4 ± 5.8	54.4 ± 3.8

Values are presented as means ± standard deviation.
